# Plasma Concentrations of Rosmarinic Acid in Patients on Antiretroviral Therapy: In Silico Exploration Based on Clinical Data

**DOI:** 10.3390/ijms25042230

**Published:** 2024-02-13

**Authors:** Maja Hitl, Nebojša Pavlović, Snežana Brkić, Gordana Dragović, Branislava Srđenović-Čonić, Nebojša Kladar

**Affiliations:** 1 Department of Pharmacy, Faculty of Medicine, University of Novi Sad, 21000 Novi Sad, Serbia; nebojsa.pavlovic@mf.uns.ac.rs (N.P.); branislava.srdjenovic-conic@mf.uns.ac.rs (B.S.-Č.); nebojsa.kladar@mf.uns.ac.rs (N.K.); 2 Center for Medical and Pharmaceutical Investigations and Quality Control, Faculty of Medicine, University of Novi Sad, 21000 Novi Sad, Serbia; 3 Department of Infectious Diseases, Faculty of Medicine, University of Novi Sad, 21000 Novi Sad, Serbia; snezana.brkic@mf.uns.ac.rs; 4 Clinic for Infectious Diseases, Clinical Centre of Vojvodina, 21000 Novi Sad, Serbia; 5 Department of Pharmacology, Clinical Pharmacology and Toxicology, Faculty of Medicine, University of Belgrade, 11000 Belgrade, Serbia; gordana.dragovic@med.bg.ac.rs

**Keywords:** rosmarinic acid, lemon balm, HIV, efavirenz, darunavir, raltegravir, plasma concentrations

## Abstract

Rosmarinic acid (RA) is a phenolic compound with antiviral properties, often encountered in dietary supplements and herbal drugs. Data on the pharmacokinetics of RA are lacking in cases of the chronic use of supplements containing this compound, and only limited data on the metabolism and distribution of RA are available. The aim of the study was to investigate the plasma levels of RA after 12 weeks of use and determine potential interactions of RA and selected antiretroviral drugs. Patients infected with human immunodeficiency virus took a supplement containing RA for 12 weeks, after which the RA concentrations in the plasma samples were analyzed. A detailed in silico analysis was conducted in order to elucidate the potential interactions between RA and the drugs efavirenz, darunavir and raltegravir. It was found that RA can be detected in patients’ plasma samples, mainly in the form of sulphoglucuronide. The potential interactions are suggested on the level of liver metabolizing enzymes and efflux P-glycoprotein, with RA competing with antiretroviral drugs as a substrate in metabolism and distribution systems. The present study suggests that the simultaneous use of RA and antiretroviral therapy (containing efavirenz, darunavir or raltegravir) may affect the plasma levels of RA after prolonged supplementation.

## 1. Introduction

Rosmarinic acid (RA) represents a phenolic compound, an ester of caffeic acid and (R)-(+)-3-(3,4-dihydroxyphenyl)lactic acid. The compound is commonly encountered in Boraginaceae and Lamiaceae plants, such as borage, mint, lemon balm, rosemary, lavender, thyme, basil, sage, savory and many others. Besides being present in herbs of culinary importance, RA is also frequently used in herbal medicines, herbal dietary supplements and natural cosmetics [1,2,3]. Numerous studies have highlighted the importance and therapeutic potential of this compound [4,5,6,7]. So far, clinical studies have shown the benefit of its use in diseases and conditions such as osteoarthritis [8], atopic dermatitis [9], allergic rhinoconjuctivitis [10,11] and metabolic syndrome [12,13,14,15] as well as a substance improving cognitive performance [16,17,18]. Additionally, phytotherapy recognizes plants with a high content of RA as antiviral agents [19]. The main support of such claims often comes from the traditional and topical use of lemon balm as a medicinal herb used in the treatment of *herpes labialis* [20,21]. Some in vitro studies have suggested the potential of RA and its derivatives against human immunodeficiency virus (HIV) [22]. However, there are still some knowledge gaps that prevent the further use of RA-containing phytopreparations. Studies of the peroral use of RA drugs and supplements in humans are less frequent and usually report the plasma concentrations only after a single dose of RA [23,24] and rarely after prolonged use [25]. Additionally, data on the effects of RA on human liver enzymes and P-glycoprotein and its interaction(s) with conventional drugs are still limited [26].

In silico methods have been widely used in all steps of the drug discovery process, from the initial phases, such as target identification and validation, to lead optimization, which filters out the biomolecules with a potentially low pharmacological profile and generally accelerates the discovery of new drugs [27,28]. In addition, systems pharmacology approaches have been recently used to identify active natural products and investigate their mechanisms of action, providing a new strategy for discovering novel drug combinations for the treatment of complex diseases [29]. Using computer-assisted in silico approaches, it is possible to identify the most promising pharmacological targets by means of network pharmacology methods to predict various biological activities based on the structural formulas to analyze different protein–ligand interactions and identify the putative molecular mechanisms of toxicity and side effects of drugs [30]. By studying the interactions of drug molecules with metabolizing enzymes and membrane transport proteins, in silico methods can be used to predict and explain drug–drug interactions, which should result in the design of safer and more effective drug therapies [31]. 

The aim of our study was to investigate the impact of three antiretroviral drugs on the pharmacokinetic profile of RA in patients infected with HIV as well as elucidate the mechanisms responsible for pharmacokinetic interactions between RA and antiretroviral drugs.

## 2. Results

### 2.1. Lemon Balm Extract and Quality Control Check

A commercially available dietary supplement containing lemon balm leaf extract was subjected to quality control. Using the high-performance liquid chromatography (HPLC) method, it was found that the declared content of RA corresponded to the detected content of the compound in this dietary supplement (111.81 ± 2.49%). The chromatogram of the analyzed dietary supplement is given in Figure S1.

### 2.2. Plasma Concentrations of Rosmarinic Acid

**Figure 1:**
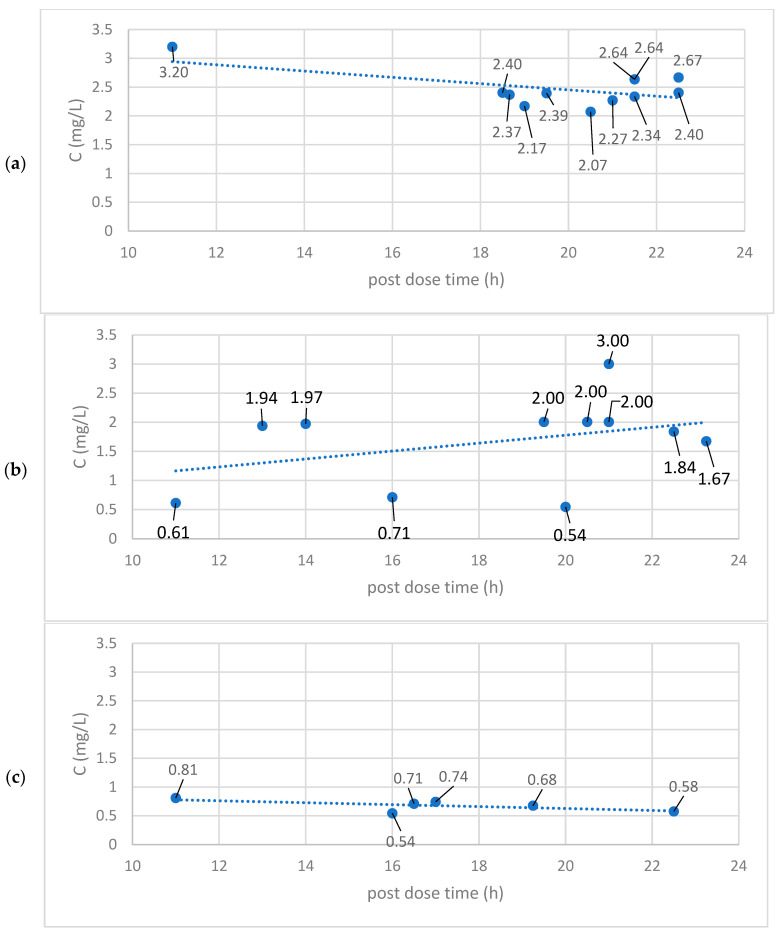
Plasma concentrations (C, in mg/L) of rosmarinic acid at corresponding post-dose time (h) in (**a**) efavirenz (n = 12), (**b**) darunavir (n = 11) and (**c**) raltegravir (n = 6) patient groups, with n being the number of patients in each group.

After 12 weeks of lemon balm extract consumption, blood samples were taken from patients and the plasma was analyzed for RA content. Bearing in mind that RA was the most abundant compound in the used dietary supplement, it was assumed that this compound was the active ingredient and that it could be used as a plasma marker of lemon balm use. Figure 1a–c represent the plasma concentrations of RA (expressed as mg/L of plasma, on y-axis) in the blood samples of each single patient for the efavirenz, darunavir and raltegravir groups of patients, respectively, dependent on the amount of time that passed since the last dose of lemon balm extract was taken (post-dose time, on x-axis).

An example of a chromatogram of an analyzed plasma sample is given in Figure S2. 

Based on previously published data on the metabolism of RA, it was assumed that after the longer period (12 weeks) of lemon balm extract intake, the compound would be detected in the conjugated form. The initial analysis of the patients’ blood samples, without pre-treatment with the enzyme (β-glucuronidase with side-activity of sulphatase), failed to detect RA. This result suggested that RA can be encountered in low concentrations in its main form and that sulpho- and glucuronide forms are the main metabolites after prolonged intake of dietary supplements containing RA. 

### 2.3. In Silico Prediction of Interactions between Rosmarinic Acid and Antiretroviral Drugs

The estimated activities of the tested compounds were predicted as the probable activity (Pa) and the probable inactivity (Pi) using the PASS online server, and only the activities with Pa > Pi were taken into account. Ten pharmacological activities with the highest probability for each investigated substance are shown in Table 1. Rosmarinic acid was mainly predicted as a compound improving membrane integrity, as an antioxidant compound with antihypoxic and antidiabetic activities, among others. As expected, efavirenz, darunavir and raltegravir were predicted to exert antiviral activity against HIV, but they were also demonstrated to interact with different CYP isoforms as well as with other enzymes.

Using the PASS server, RA was predicted to act as a substrate of different CYP isoforms. CYPs where Pa > 0.3 and Pa/Pi > 2 were considered as potential metabolizing enzymes for RA and 15 CYP isoforms were identified. Efavirenz, darunavir and raltegravir were then tested to assess whether they could interact (act as substrate, inducer or inhibitor) with these CYP isoforms potentially involved in the metabolism of RA. Efavirenz was predicted to interact with four CYP isoforms (CYP4A11, CYP4A, CYP3A2, CYP3A4), darunavir with three (CYP2H, CYP2C, CYP3A4) and raltegravir with only one CYP isoform (CYP2H) (Table 2).

Using the Vienna LiverTox web service, RA was tested for potential interactions with membrane transport proteins that affect drug tissue distribution. Rosmarinic acid was predicted to act as a P-glycoprotein ligand. Efavirenz, darunavir and raltegravir were then tested to assess whether they could interact with the same transporters potentially involved in the distribution of RA. Darunavir was predicted to act as a P-glycoprotein substrate, while raltegravir was predicted to inhibit this efflux transporter. The interactions of the tested compounds with P-glycoprotein and other membrane transport proteins are presented in Table 3.

**Figure 2:**
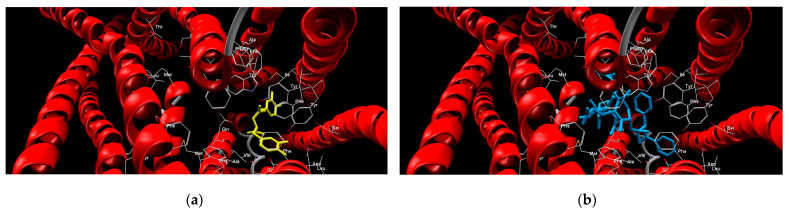
Molecular docking of (**a**) rosmarinic acid and (**b**) paclitaxel at the binding site of P-glycoprotein.

The investigated compounds, along with paclitaxel as a control ligand, were subjected to molecular docking analyses, and the binding affinity values are shown in Table 4. The ligand–protein interactions were analyzed through the values of the MolDock Scores. The MolDock scoring function is derived from the piecewise linear potential (PLP) scoring functions improved with a new hydrogen bonding term and new charge schemes. As expected, the interaction energy was highest for paclitaxel (−204.484 kcal mol^−1^), while efavirenz showed the lowest affinity towards the P-glycoprotein binding site (−97.532 kcal mol^−1^). The poses of rosmarinic acid and paclitaxel forming the most stable complexes with P-glycoprotein are shown in Figure 2, respectively. The electrostatic and steric interactions mostly contributed to the stabilization of both the paclitaxel- and RA-protein complexes, with similar hydrogen bonding energies of approximately 5.2 kcal mol^−1^. 

## 3. Discussion

As previously stated, RA was used as a marker of lemon balm use. To the best of our knowledge, this is the first investigation to detect an RA concentration in blood after the longest period during which a herbal extract containing this compound was used. Previously, the longest duration of usage of such a herbal extract, alongside the detection of metabolites, was documented as 30 days [25]. Other earlier studies mainly opted for analyzing the RA concentration in plasma after an intake of a single dose of herbal extract containing this compound [3]. While investigating the pharmacokinetic properties of RA in humans, an extract of *Perilla frutescens* (L.) Britton, Lamiaceae was used in one study; this study demonstrated the presence of RA (as well as other metabolites, such as methyl-RA and ferulic acid) in conjugated forms, mainly suplhoglucuronide. The plasma concentration of RA (both conjugated and non-conjugated forms) detected after the peroral intake of 200 mg of *P. frutescens*’ extract was 1.15 ± 0.28 μmol/L [23]. Another study compared the peroral intake of smaller and larger doses. It was found that doubling the dosage of lemon balm extract (from 250 mg to 500 mg of lemon balm extract) led to an approximate doubling of the RA concentration in the plasma (from 72.22 ± 12.01 nmol/L to 162.20 ± 40.20 nmol/L). However, it was also found that an increase in the dosage also resulted in elevated concentrations of non-conjugated forms, indicating saturational kinetics of conjugation [24]. This finding is in contrast with the study presented here, which suggests that the prolonged usage (12 weeks) of higher doses of herbal extract (600 mg per day) containing RA results in higher concentrations of RA in conjugated forms. Furthermore, this suggests that the metabolism of the compound can be directed towards the conjugation processes and/or that chronic administration increases the activity of enzymes involved in this phase of the metabolism. Similar to this, the extended usage (30 days) of spearmint (*Mentha spicata* L., Lamiaceae) failed to detect the primary form of RA in blood (with the main detected metabolite being glucuronide of methyl-RA) [25].

According to the literature, studies investigating pharmacokinetic interactions between antiretroviral drugs and medicinal herbs typically do not assess the concentrations of presumed active principles of these herbs in patients’ blood. In cases where pharmacokinetic interactions are confirmed, determining the concentrations of the active compounds in plasma/blood is useful for determining the doses which lead to interactions as well as the future management of therapy during concomitant use. Furthermore, it is important to elucidate the mechanism(s) leading to these interactions in order to manage any other potential interactions mediated via the same mechanism.

Pharmacokinetic clinical data on plasma concentrations of RA in patients on antiretroviral therapy, which were obtained in the present study, can be explained by using several computational approaches. Firstly, through using a PASS online server, our study predicted that RA primarily enhances membrane integrity while exerting antioxidant, antihypoxic, antidiabetic and anticarcinogenic activities. It was previously demonstrated using inverse docking methodology that RA shows binding to proteins involved in carcinogenesis, specifically matrix metalloproteinase-3 and phospholipase A2, which might contribute to its strong anti-inflammatory activity. According to the results of the same study, RA may form a strong complex with coagulation factor X and reduce excessive blood clotting as well as exert antiparasitic activity following its binding to farnesyl pyrophosphate synthase [32].

Using the same in silico approach, RA was predicted to be metabolized via the CYP450 hepatic enzyme complex, and 15 CYP isoforms were identified as potential metabolizing enzymes for RA. While none of the studied antiretroviral drugs were estimated to induce or inhibit any of the CYP isoforms potentially involved in the metabolism of RA, they were predicted to act as substrates for certain CYP isoforms. Efavirenz was predicted to interact with CYP4A11, CYP4A, CYP3A2 and CYP3A4, darunavir with CYP2H, CYP2C and CYP3A4 and raltegravir only with CYP2H. The co-administration of drugs that compete as substrates for the same individual CYPs may increase their plasma concentrations [33]. This is in accordance with our clinical data since the highest plasma concentrations of RA were measured in patients taking efavirenz, while the lowest ones were in patients taking raltegravir.

The potential interactions between RA and antiretroviral drugs were also investigated at a transporter level using a computational approach. Rosmarinic acid was predicted in this study to act as a substrate of P-glycoprotein. This is in accordance with a previous study that demonstrated, using molecular docking, that RA may bind to P-glycoprotein and serve as a substrate [34]. Furthermore, RA radiolabeled with ^99m^Tc was predicted to be a substrate of P-glycoprotein as well [35]. P-glycoprotein, as a member of ATP-binding cassette (ABC) transporters, is constitutively expressed in the apical membrane of many epithelial and endothelial barriers, such as the blood–brain barrier, the blood–testicular barrier and the blood–intestinal barrier, preventing the distribution of numerous drugs, including several antiretroviral drugs, in these tissues [36]. Therefore, nanotherapeutic strategies with P-glycoprotein efflux inhibitors have been extensively investigated as a Trojan horse approach to enhancing intracellular concentrations of antiretroviral drugs that are P-glycoprotein substrates, ultimately improving HIV infection eradication. [37].

Using the Vienna LiverTox web service, darunavir was predicted to act as a P-glycoprotein substrate, raltegravir to inhibit P-glycoprotein, and efavirenz not to act as either a substrate or an inhibitor of this efflux transporter. The obtained results may explain the highest plasma concentrations of RA, as a P-glycoprotein substrate, in patients taking efavirenz and the lowest in patients taking raltegravir. The results of our in silico analysis are in accordance with the results of several in vitro and ex vivo studies. Investigating the effects of efavirenz on intestinal P-glycoprotein function on everted gut sac and *in situ* intestinal perfusion rat models, using rhodamine 123 as P-glycoprotein substrate, Berruet et al. [38] showed that repeated oral doses of efavirenz did not modulate intestinal P-glycoprotein activity. Similarly, Mouly et al. [39] did not demonstrate any induction of intestinal P-glycoprotein expression in human intestinal biopsies following a 10-day treatment course with efavirenz. On the other hand, darunavir was at first found to be a P-glycoprotein substrate in human intestinal and renal cell lines [40], suggesting competition with RA for P-glycoprotein and a subsequent decrease in its efflux. Furthermore, Holmstock et al. [41] confirmed that P-glycoprotein has a modulatory effect on the absorption of darunavir using an in situ intestinal perfusion technique in P-gp knockout mice, even at a relevant intraluminal concentration of this drug. Lastly, raltegravir transport by P-glycoprotein remains controversial. Most previous in vitro and ex vivo studies suggested that raltegravir was a substrate for P-glycoprotein [42,43]. However, Dupuis et al. [44] reported that raltegravir may behave as a weak substrate for P-glycoprotein, but it shows no interactions with P-glycoprotein in a monoclonal antibody UIC2 shift test, as a sensitive assay to analyze conformational transition associated with P-glycoprotein function. These results suggest that different experimental methods should be used to unequivocally confirm the interactions with membrane transport proteins.

The possible underlying mechanisms of the obtained clinical data can also be explained in terms of the in silico binding affinities towards P-glycoprotein, which is potentially involved in RA distribution. Efavirenz had the lowest affinity towards P-glycoprotein in our molecular docking analysis, which is in accordance with other in silico analysis results and clinical data. Raltegravir showed affinity to the studied binding site of P-glycoprotein in molecular docking analysis, although LiverTox analysis indicated it acts as an inhibitor rather than a substrate. However, it should be noted that most of the P-glycoprotein inhibitors interact with the same binding site as the P-glycoprotein substrates, indicating that both classes of compounds could share several structural similarities [45]. Additionally, this transport protein has more than five substrate binding sites, which can transport a broad spectrum of substrates [46]. Therefore, it should be taken into account that interactions between RA and other drugs may occur within other binding sites of P-glycoprotein.

## 4. Materials and Methods

### 4.1. Lemon Balm Extract and Quality Control Check

This study used commercially available lemon balm (*Melissa officinalis* L., Lamiaceae) leaf extract, standardized at a minimum of 7% RA. This extract is formulated as capsules and registered as a dietary supplement. In order to validate the content of RA, a previously described analytical method was applied [47]. In brief, high-performance liquid chromatography (HPLC-DAD) was performed (Agilent HP 1100 HPLC coupled with diode array detector - DAD); separation of the components was achieved on a Nucleosil C18 (5 μm × 4.6 mm × 250 mm) column heated to 30 °C. A gradient elution was applied, with 1% formic acid being mobile phase A and methanol being mobile phase B; the program of elution was as follows: 0–10 min, 10–25% B; 10–20 min, 25–45% B; 20–30 min, 45% B; 30–35 min, 45–70% B; 35–40 min, 70–100% B; 40–43 min, 100% B. The chromatogram of RA was monitored at 330 nm. The result is expressed as a percentage of the declared content.

### 4.2. Patients

Patients with HIV infection, formally treated in the Center for HIV/AIDS at the Clinic for Infectious Diseases of the Clinical Center of Vojvodina, participated in this study. The study included male patients (>18-years-old) with an undetectable viral load (˂50 copies of HIV RNA/mL of plasma) during the previous year or more, with an unchanged antiretroviral regimen during the previous 6 months or more. Additionally, the patients were excluded in the case of the use of conventional drugs that can alter the pharmacokinetics of antiretroviral drugs, in the case of the use of other herbal medicines and/or supplements, in the case of co-infection with hepatitis B and/or C virus(es), in the case of liver diseases (e.g., fibrosis, cirrhosis. etc.,) and in the case of allergy or other types of sensitivity to lemon balm or RA. The patients were invited to participate on a volunteer basis, and Informed Consent was obtained before the start of the study in written form. The patients were nominally divided into three groups according to the drug contained in the antiretroviral treatment regimen: 1. efavirenz (12 patients), 2. darunavir (11 patients) and 3. raltegravir (6 patients) (these being drugs added to standard “backbone of therapy”, consisted of nucleoside inhibitors of reverse transcriptase). Any required medical data of the patients were obtained from their standard medical charts, held at the Clinic of Infectious Diseases.

### 4.3. Study Design

The study was designed as a prospective, interventional, open clinical study. The patients received an adequate amount of lemon balm extract (formulated as a capsule) before the start of the study. The instruction given to them was to take two capsules (corresponding to 600 mg of lemon balm extract) with one half of a glass of water, approximately half an hour before night-time sleep. The patients took this supplement for 12 weeks. During the entire time, there were no changes to the antiretroviral drug therapy nor its regimen. The details of the patients’ antiretroviral therapies are given in Table S1.

### 4.4. Blood Samples and Plasma Analysis

At the end of the study, blood samples were taken from the patients. The blood was sampled after more than 10 h after administering the last dose of lemon balm extract. The samples were centrifuged immediately after being taken, and the citrate plasma was frozen and kept at −20 °C until further preparation and analysis.

The plasma preparation before the analysis included treatment of the plasma according to previously published research, with minor modifications [24]. In brief, the plasma samples were treated with the enzyme β-glucuronidase, originating from the Roman snail (*Helix pomatia* L., Helicidae) (Sigma Aldrich, St. Louis, MO, USA). Besides the main activity of the deglucuronidation of RA, this enzyme performs a side-activity of deconjugating sulpho-groups. The plasma samples were treated with the enzyme solution at a ratio of 1:1 and incubated at 37 °C for 45 min. The reaction was stopped using acetic acid in methanol, with the plasma proteins being precipitated, and the entire contents of the test tube were centrifuged. The supernatant was filtrated and used for further analysis.

The analysis was performed according to previously published research, with minor modifications [48]. In brief, a HPLC method was performed (Agilent HP 1100 HPLC coupled with DAD). Separation of the components was performed on a Nucleosil C18 (5 μm × 4.6 mm × 250 mm) column kept at room temperature (25 °C). A gradient elution was performed, with methanol being mobile phase A and a 0.1% (m/m) aqueous solution of phosphoric acid being mobile phase B; the program of elution was as follows: 0–5 min, 55% B; 5–10 min, 20% B. The chromatograms of RA were monitored at 330 nm. The results were expressed as mg/L of plasma.

### 4.5. Computational Analysis

The 2D chemical structures of the investigated drug molecules (RA, efavirenz, darunavir and raltegravir) were drawn using ChemDraw Professional 16.0 (PerkinElmer, Waltham, MA, USA), and the simplified molecular-input line-entry system (SMILES) was utilized to conduct the computational analysis (PASS online and Vienna LiverTox web servers). For the molecular docking analyses, 2D structures of the compounds were converted to 3D using Chem3D 16.0 (PerkinElmer, Waltham, MA, USA), and their geometry was optimized using the semi-empirical MM2 method. The conformation that was found to be the most stable was determined and stored in mol2 form [49].

#### 4.5.1. Prediction of Activity Spectra for Substances (PASS)

The PASS online server (https://www.way2drug.com, accessed on 17 December 2023) was used for the prediction of the pharmacological activities for the investigated drugs. The activity spectrum estimation algorithm uses a Bayesian method, and the probability of being active (Pa) to the probability of being inactive (Pi) ratios were calculated. According to leave-one-out cross-validation (LOOCV) estimation, the average prediction accuracy is around 95%. Only actions with Pa > Pi were considered feasible for the tested drugs. Pa > 0.7 indicated a high probability of experimental pharmacological effect, Pa between 0.5 and 0.7 indicated a moderate probability, while Pa less than 0.5 indicated negligible pharmacological activity [50].

#### 4.5.2. Prediction of Interactions with Transport Proteins

A publicly available Vienna LiverTox web service was used to predict the interaction profiles of RA, efavirenz, darunavir and raltegravir with transporters potentially involved in the body distribution of RA, i.e., to predict whether the investigated compounds were substrates/inhibitors of P-glycoprotein, BCRP, BSEP and MRP3 transporters [51]. This web service predicts a binary outcome, indicating whether the query compound is active or not. It contains a set of machine learning models for the prediction of interaction profiles of small molecules with transporters relevant for regulatory agencies. Each transporter model is based on a different classifier, and the structure of the molecules is described with RDKit descriptors [52]. A detailed description of the models is given at https://livertox.univie.ac.at [53] (accessed on 17 December 2023).

#### 4.5.3. Molecular Docking Analysis

Docking studies were performed using Molegro Virtual Docker (MVD) software, version 6.0 [54]. The three-dimensional (3D) crystal structure of P-glycoprotein in a complex with paclitaxel (PDB code: 6QEX) [55], which has been determined by electron microscopy at a resolution of 3.60 Å, was retrieved from the Protein Data Bank.

The investigated compounds in mol2 format and P-glycoprotein in pdb format were imported into the MVD program. All the solvent molecules were removed from the protein structure. The docking protocol was validated by “redocking” the co-crystalized ligand, and the root mean square distance (RMSD) of the docked ligand was within the reliable range of 2 Å, so it was verified after the docking protocol that paclitaxel could interact with the crystal structure of 6QEX similarly to the preexisting co-crystallized paclitaxel. The potential ligand binding sites for P-glycoprotein were predicted using MVD, and the cavity containing the co-crystallized paclitaxel was selected as the active site for further docking analyses. Different orientations of the ligands were searched and ranked based on their energy scores. All the docking calculations were carried out using the grid-based MolDock score function with a grid resolution of 0.30 Å. The binding site on the receptor was defined as a spherical region that encompasses all protein atoms within 10 Å of the crystallographic ligand molecule. MolDock SE was used as a search algorithm and the number of runs was set to 10. A population size of 50 and a maximum iteration of 1500 were used for the parameter settings. The number of generated poses was 5, and the best pose of each compound was selected for the subsequent ligand–protein interaction energy analysis.

## 5. Conclusions

The prolonged use of supplements containing rosmarinic acid results in detectable concentrations in the plasma of patients on antiretroviral therapy, with RA being encountered in the form of sulphoglucuronide. The concentrations were highest in patients taking efavirenz and the lowest in patients of the raltegravir group. These differences can be attributed to both rosmarinic acid and efavirenz being substrates for the same liver enzymes involved in metabolism and also to competition in efflux P-glycoprotein, with rosmarinic acid and darunavir both being substrates for it. The results of this study suggest that the concomitant use of rosmarinic acid-containing phytopreparations and antiretroviral therapy (containing one of the drugs efavirenz, darunavir or raltegravir) may result in altered plasma concentrations of rosmarinic acid in these patients.

## Figures and Tables

**Table 1:** Prediction of activity spectra for investigated compounds.

Compound	Pa	Pi	Activity
**Rosmarinic acid** 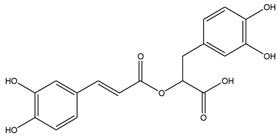	0.956	0.003	Membrane integrity agonist
	0.938	0.003	Feruloyl esterase inhibitor
	0.921	0.002	Antihypoxic
	0.836	0.003	Monophenol monooxygenase inhibitor
	0.799	0.005	Antidiabetic
	0.804	0.020	CYP2J substrate
	0.787	0.011	GST A substrate
	0.779	0.004	Reductant
	0.785	0.012	Membrane permeability inhibitor
	0.766	0.004	Pyruvate decarboxylase inhibitor
**Efavirenz** 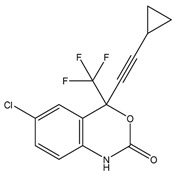	0.898	0.003	Antiviral (HIV)
	0.881	0.004	Antiviral
	0.879	0.001	HIV-1 reverse transcriptase inhibitor
	0.853	0.003	Biliary tract disorder treatment
	0.711	0.071	Phobic disorder treatment
	0.600	0.005	Skeletal muscle relaxant
	0.589	0.005	DNA directed RNA polymerase inhibitor
	0.556	0.017	Prostate disorder treatment
	0.521	0.041	CYP3A1 substrate
	0.490	0.016	Muscle relaxant
**Darunavir** 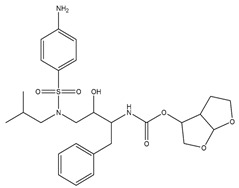	0.878	0.003	Antiviral (HIV)
	0.873	0.004	Antiviral
	0.834	0.002	HIV-1 protease inhibitor
	0.537	0.051	CYP3A substrate
	0.464	0.021	P-glycoprotein substrate
	0.539	0.106	CDP-glycerol phosphotransferase inhibitor
	0.477	0.067	CYP3A4 substrate
	0.423	0.024	CYP2A11 substrate
	0.402	0.040	CYP2C19 inducer
	0.412	0.100	Anaphylatoxin receptor antagonist
**Raltegravir** 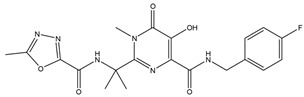	0.641	0.002	HIV-1 integrase inhibitor
	0.528	0.006	Antiviral
	0.463	0.004	Antiviral (HIV)
	0.440	0.003	Poly(ADP-ribose) polymerase 2 inhibitor
	0.424	0.059	PDGF receptor kinase inhibitor
	0.288	0.005	Gastrointestinal disorder treatment
	0.323	0.045	DNA polymerase I inhibitor
	0.287	0.045	Thiol protease inhibitor
	0.282	0.046	Raynaud’s phenomenon treatment
	0.241	0.053	Antineoplastic enhancer

Pa–probable activity, Pi–probable inactivity, CYP–cytochrome P450, GST–glutathione S-transferase, HIV–human immunodeficiency virus, DNA–deoxyribonucleic acid, RNA–ribonucleic acid, CDP–cytidine diphosphate, PDGF–platelet-derived growth factor.

**Table 2:** Impact of efavirenz, darunavir and raltegravir on different CYP isoforms that were predicted to be involved in rosmarinic acid metabolism.

CYPs Metabolizing Rosmarinic Acid	Influence of Efavirenz	Influence of Darunavir	Influence of Raltegravir
CYP2J (Pa 0.804, Pi 0.020)	/	/	/
CYP2J2 (Pa 0.693, Pi 0.035)	/	/	/
CYP4A11 (Pa 0.553, Pi 0.012)	substrate (Pa 0.311, Pi 0.122)	/	/
CYP4A (Pa 0.534, Pi 0.008)	substrate (Pa 0.346, Pi 0.051)	/	/
CYP2C12 (Pa 0.516, Pi 0.086)	/	/	/
CYP2C8 (Pa 0.411, Pi 0.063)	/	/	/
CYP2F1 (Pa 0.373, Pi 0.063)	/	/	/
CYP26A (Pa 0.303, Pi 0.004)	/	/	/
CYP2H (Pa 0.446, Pi 0.167)	/	substrate (Pa 0.424, Pi 0.190)	substrate (Pa 0.394, Pi 0.223)
CYP2D16 (Pa 0.358, Pi 0.098)	/	/	/
CYP2C6 (Pa 0.307, Pi 0.049)	/	/	/
CYP2A4 (Pa 0.324, Pi 0.093)	/	/	/
CYP2C (Pa 0.307, Pi 0.116)	/	substrate (Pa 0.351, Pi 0.094)	/
CYP3A2 (Pa 0.353, Pi 0.177)	substrate (Pa 0.453, Pi 0.104)	/	/
CYP3A4 (Pa 0.306, Pi 0.146)	substrate (Pa 0.390, Pi 0.093)	substrate (Pa 0.477, Pi 0.067)	/

CYP-cytochrome P450, Pa-probable activity, Pi-probable inactivity, /-impact not determined.

**Table 3:** Prediction of interactions between investigated compounds and membrane transport proteins.

	Rosmarinic Acid	Efavirenz	Darunavir	Raltegravir
P-glycoprotein				
transport	positive	negative	positive	negative
inhibition	negative	negative	negative	positive
BCRP				
transport	positive	positive	negative	positive
inhibition	negative	positive	negative	negative
BSEP				
transport	negative	positive	positive	negative
inhibition	negative	negative	positive	positive
MRP3				
transport	negative	negative	negative	negative
inhibition	negative	negative	positive	positive

BCRP-breast cancer resistance protein, BSEP-bile salt export pump, MRP3-multidrug resistance-associated protein 3.

**Table 4:** Molegro binding energies (kcal mol^−1^) of best binding poses for ligands in P-glycoprotein.

Ligand	MolDock Score (kcal mol^−1^)
Rosmarinic acid	−114.253
Efavirenz	−97.532
Darunavir	−157.616
Raltegravir	−126.506
Paclitaxel	−204.484

## Data Availability

The data obtained in this study are fully available in the main text and Supplementary Material of this article.

## Supplementary Materials

The following supporting information can be downloaded at https://www.mdpi.com/article/10.3390/ijms25042230/s1.
