# Supplementary material for: Plasma Concentrations of Rosmarinic Acid in Patients on Antiretroviral Therapy: In Silico Exploration Based on Clinical Data
Source: Int J Mol Sci. 2024 Feb 13;25(4):2230. doi: 10.3390/ijms25042230 (PMC10888967; doi:10.3390/ijms25042230)
Supplement: Supplementary file 1 [file ijms-25-02230-s001.zip › ijms-2854110-supplementary.pdf]

Supplementary Files

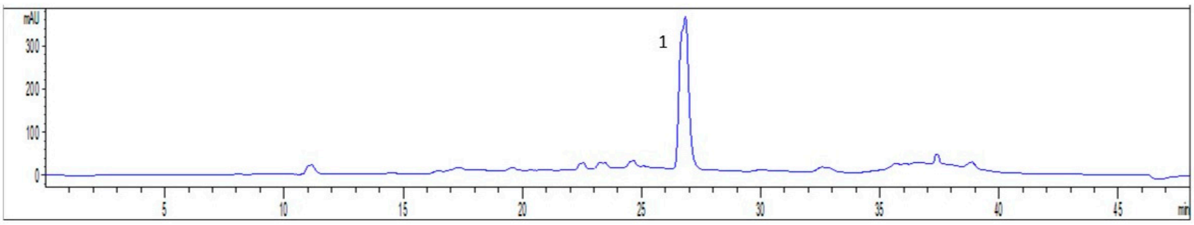

Figure S1. Chromatogram of analyzed dietary supplement containing lemon balm's extract with detection at 330 nm.

Identified compound: 1- rosmarinic acid.

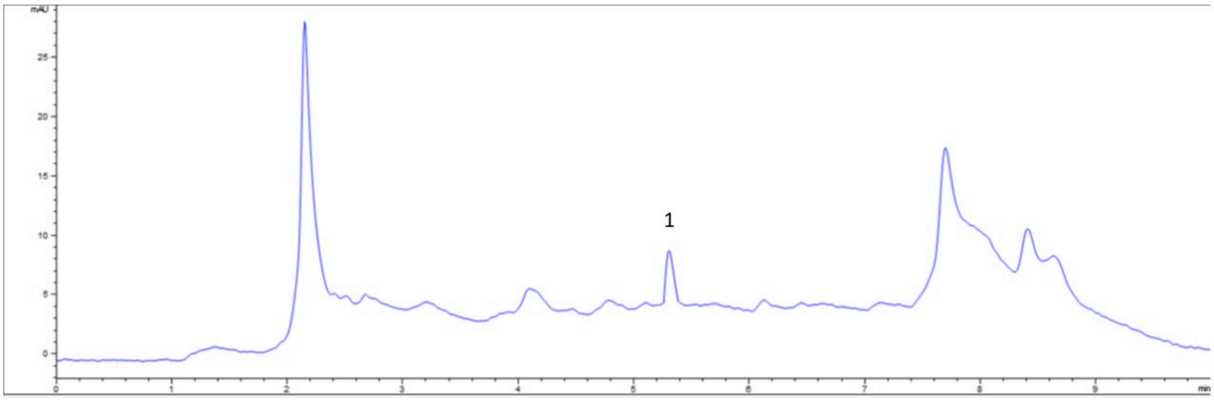

Figure S2: An example chromatogram of analyzed plasma sample (patient of darunavir group, code DRV+MAT-1) with detection at 330 nm.

Identified compound: 1- rosmarinic acid.

Table S1: Antiretroviral drugs and regimen of patients included in study.

Patient's code	Antiretroviral drugs (brand name)[1]
	Efavirenz group
MAT+EFV-1	Truvada, Stocrin
MAT+EFV-2	Truvada, Stocrin
MAT+EFV-3	Kivexa, Stocrin
MAT+EFV-5	Kivexa, Stocrin
MAT+EFV-6	Kivexa, Stocrin
MAT+EFV-7	Kivexa, Stocrin
MAT+EFV-8	Truvada, Stocrin
MAT+EFV-9	Kivexa, Stocrin
MAT+EFV-10	Kivexa, Stocrin
MAT+EFV-12	Kivexa, Stocrin
MAT+EFV-13	Kivexa, Stocrin
MAT+EFV-14	Kivexa, Stocrin
	Darunavir group
MAT+DRV-1	Truvada, Prezista, Norvir
MAT+DRV-3	Kivexa, Rezolsta
MAT+DRV-4	Gilestra duo, Prezista, Norvir
MAT+DRV-5	Gilestra duo, Rezolsta
MAT+DRV-6	Truvada, Rezolsta
MAT+DRV-7	Kivexa, Rezolsta
MAT+DRV-8	Gilestra duo, Rezolsta
MAT+DRV-13	Truvada, Rezolsta
MAT+DRV-14	Kivexa, Rezolsta
MAT+DRV-15	Gilestra duo, Rezolsta
MAT+DRV-17	Gilestra duo, Rezolsta
	Raltegravir group
MAT+RAL-3	Gilestra duo, Isentress
MAT+RAL-4	Gilestra duo, Isentress
MAT+RAL-6	Truvada, Isentress
MAT+RAL-9	Truvada, Isentress
MAT+RAL-11	Gilestra duo, Isentress
MAT+RAL-12	Truvada, Isentress

[1] The drugs are presented with trademark names. These drugs contain the following active substances:

Truvada – emtricitabine, tenofovir disoproxil fumarate (taken once daily)
Kivexa – abacavir sulphate and lamivudine (taken once daily)
Gilestra duo – tenofovir disopoxil fumarate, emtricitabin (taken once daily)
Stocrin – efavirenz (taken once daily)
Prezista – darunavir (taken once or twice daily)
Rezolsta –darunavir, cobicistat (taken once daily)
Norvir – ritonavir (taken once or twice daily)
Isentress – raltegravir (taken once daily)
